# Aberrant signaling in tonsillar B cells producing pathogenic *O*-glycoforms of IgA1 in IgA nephropathy

**DOI:** 10.3389/fimmu.2026.1737992

**Published:** 2026-01-30

**Authors:** Koshi Yamada, Kei Ogiwara, Jan Novak, Yusuke Suzuki

**Affiliations:** 1 Department of Nephrology, Juntendo University Faculty of Medicine, Tokyo, Japan; 2 Department of Microbiology, University of Alabama at Birmingham, Birmingham, AL, United States

**Keywords:** a proliferation-inducing ligand (APRIL), galactose-deficient IgA1 (Gd-IgA1), genome-wide association studies (GWAS), IgA nephropathy, leukemia inhibitory factor (LIF), nasal-associated lymphoid tissue (NALT), toll-like receptor 9 (TLR9), tonsillectomy

## Abstract

IgA nephropathy (IgAN) is a mesangioproliferative glomerulonephritis characterized by IgA1-containing immune-complex deposits wherein IgA1 is enriched for galactose-deficient IgA1 (Gd-IgA1) glycoforms. IgAN pathogenesis involves mucosal immune system, as IgAN onset and activity are associated with infections of the upper-respiratory tract, i.e., synpharyngitic hematuria. Current four-hit hypothesis postulates that multiple events, starting with the production of Gd-IgA1, in genetically susceptible individuals lead to the formation of nephritogenic immune complexes and development of IgAN. Biochemical studies using IgA1-producing cell lines derived from the peripheral blood of IgAN patients and healthy controls revealed that secretion of Gd-IgA1 is due to dysregulated expression of several *O*-glycosylation enzymes. Production of Gd-IgA1 can be further upregulated by some cytokines. Genome-wide association studies identified multiple candidate genes for IgAN, serum levels of IgA, and serum levels of Gd-IgA1. Some of the IgAN-associated genes are also found in other autoimmune diseases and conditions. Notably, *HORMAD2/LIF* locus is associated with IgAN, serum levels of IgA, and tonsillectomy. In this review, we detail various findings concerning IgAN and Gd-IgA1 production by cells derived from the circulation and tonsils. Also, as tonsillectomy is commonly used in Japan as a part of treatment for IgAN, we detail biochemical and signaling studies of IgA1-producing cells derived from peripheral blood and tonsils.

## 1 Introduction

**Figure 1:**
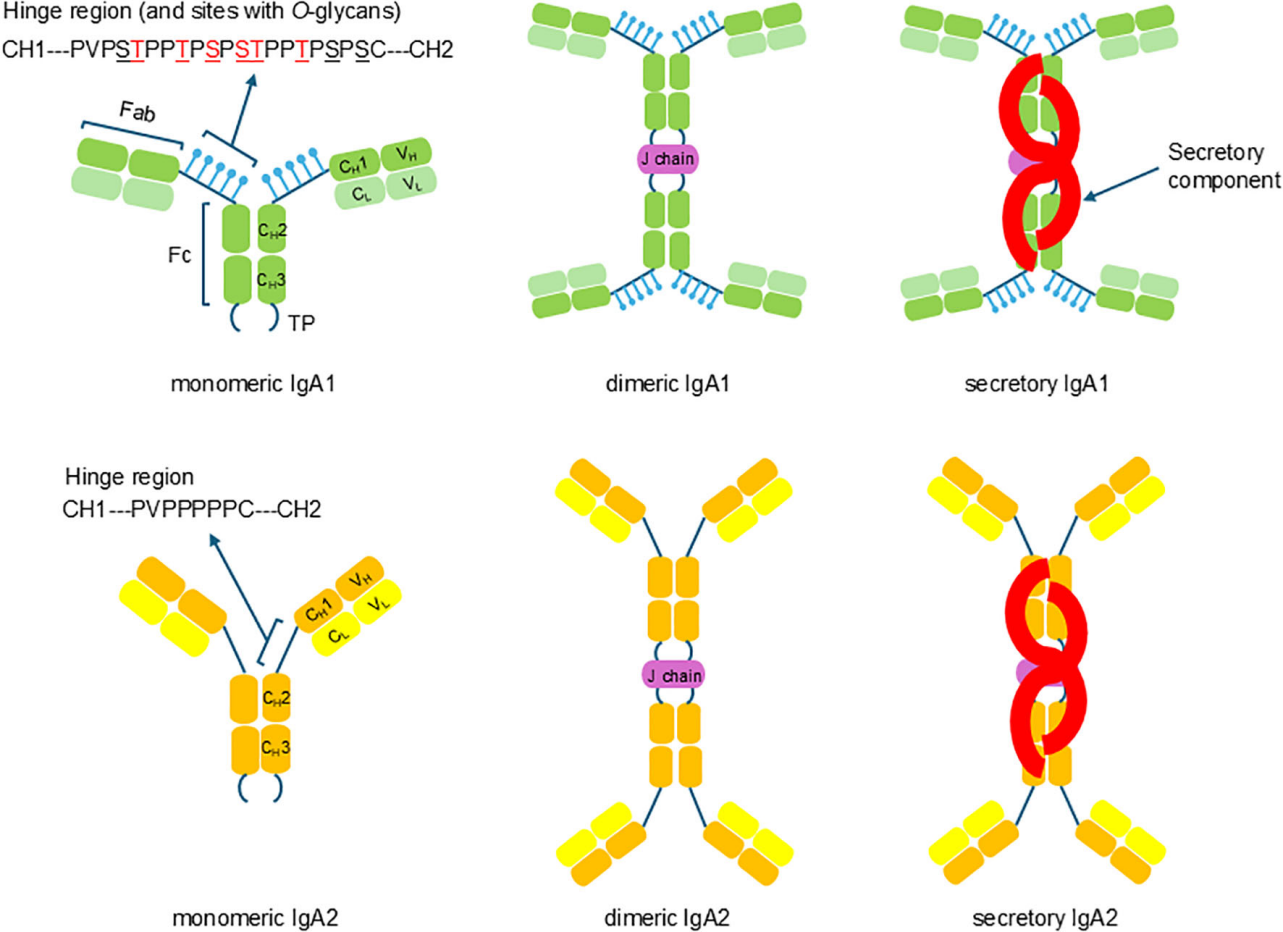
Molecular forms of human IgA1 and IgA2 (monomeric, dimeric, secretory) and their hinge-region amino-acid sequences. Comparison of amino-acid sequences of human IgA1 (top) and IgA2 (bottom) hinge regions. Human IgA1 has nine Ser (S) and Thr (T) amino-acid residues (underlined) in the hinge region segment (between constant regions CH1 and CH2 of the heavy chains). Usually, three to six clustered *O*-glycans are attached per hinge region (the six commonly utilized sites shown in red). IgA2 hinge region is shorter compared to that of IgA1, does not have Ser and Thr residues, and, thus, IgA2 does not have *O*-glycans.

The daily production of IgA is the largest of all immunoglobulin (Ig) isotypes in humans, with a daily synthesis of up to 70 mg of IgA per kg of body weight (1). IgA is secreted by IgA-producing cells in two main molecular forms (2, 3): monomeric IgA (mIgA) and dimeric IgA (dIgA). The latter has a joining chain (J-chain) that covalently connects two mIgA molecules by disulfide bridges between Cys residue in the tail segment of the IgA heavy chain and a Cys residue in J-chain. Molecular forms of J-chain-containing IgA are also called polymeric IgA, and in addition to dimeric IgA, other polymeric forms of IgA may have three or more mIgA molecules. In addition, secretory IgA (SIgA) is the main form of IgA found on mucosal surfaces; SIgA contains secretory component (SC) derived by proteolytic cleavage from the polymeric immunoglobulin receptor (PIGR) during transcytosis through mucosal epithelial cells. IgA is the second most common Ig in the peripheral blood (~2 mg/mL of serum), after IgG (~12 mg/mL of serum), and is mainly present as mIgA, with only 10% represented by dIgA. However, IgA is the most abundant Ig in external secretions on mucosal surfaces (e.g., tears, saliva, nasal secretions, gallbladder bile, and intestinal fluids and also in colostrum and milk) and is secreted locally as SIgA. Mucosal surfaces of the respiratory, gastrointestinal, and genitourinary tracts are the sites with high level of IgA secretion and also the sites used for mucosal-pathogen invasion. Important roles of IgA in the immune-defense processes are based on the abundance of IgA and its ability to form polymers (i.e., two or more mIgA molecules connected by the J-chain) that can neutralize and eliminate pathogens (4). In humans, IgA exists in two subclasses, IgA1 and IgA2. Although the two subclasses exhibit a great degree of amino-acid sequence identity, substantial differences in glycosylation impart functional differences (5). Specifically, IgA2 has five *N*-glycans per heavy chain whereas IgA1 has two *N*-glycans per heavy chain. In addition, IgA1 has clustered *O*-glycans in the extended hinge region, whereas IgA2 has a shorter hinge region without any *O*-glycans (5, 6) (**Figure 1**).

Serum IgA is produced mainly by plasma cells in the bone marrow; this IgA is predominantly a monomeric protein with a quaternary structure consisting of two heavy chains (HC) and two light chains (LC) linked by disulfide bonds. In contrast, mucosal IgA, particularly dIgA with a J chain, is produced by plasma cells close to the epithelium (7–9). Notably, involvement of mucosal-tissue-induced IgA is suspected in patients with IgA nephropathy (IgAN), as the clinical disease onset and disease activity are often associated with upper-respiratory tract infection and inflammation and the glomerular immune-complex deposits contain mostly polymeric IgA (1).

The concepts of homing and recirculation of IgA-producing cells have been emerging from various studies. It is known that IgA-producing B cells in the small intestine, including Peyer’s patches, express CCR9 and integrin α4β7 and the homing to the small intestine is impacted by retinoic acid produced by the local dendritic cells (10). Furthermore, 7α,25-dihydroxycholesterol produced by epithelial cells has additional effects of IgA-producing cells and IgA secretion (11). IgA antibodies against enterococci are detected in the feces, but not in the serum, of B6 mice kept under specific-pathogen free (SPF) conditions (12). These observations suggest that IgA-producing cells induced in the intestinal tract are usually homing to the intestinal mucosa and not to the bone marrow. The recirculation and homing are impacted by B-cell differentiation, isotype switching, responsiveness to chemoattractants, as well as cell-surface glycosylation (13). For example, a subset of B lymphocytes within secondary lymphoid organs responds to stromal cell-derived factor (SDF)-1α; this responsiveness correlates with specific localization within this lymphoid organ and is further controlled by the differentiation state of the cells and the involvement of the B-cell receptor (BCR) (14).

Genome-wide association studies (GWAS) identified multiple IgAN-associated loci, some of which are also associated with other diseases, including those characterized by disruption of the intestinal-epithelial barrier, IgA production, tonsillar infections, and abnormal responses to the gut microbiota. These findings thus support an important role for the mucosal immune responses in IgAN (15). Furthermore, the recently observed clinical improvement in IgAN patients treated with the controlled intestinal release formulation of budesonide (Tarpeyo) (16, 17), supports the notion that the pathogenic IgA in IgAN may be related to the cells originating in the intestinal mucosa. Tarpeyo is designed to be released locally in the distal ileum and proximal large intestine to decrease the production of pathogenic IgA by the IgA1-producing cell in the mucosal lymphoid tissue (Peyer’s patches). A phase III trial evaluating Tarpeyo for treatment of IgAN showed a significant reduction in short-term proteinuria and has resulted in accelerated FDA approval for use in the United States (17, 18).

In the situation when the intestinal mucosa is exposed to chronic inflammation, such as in inflammatory bowel disease, antigen-specific IgA of mucosal origin may appear in the circulation (19–21). As some patients with IgAN have a chronic inflammatory bowel disease, question arises about a possible connection between the two diseases. And, conversely, what differentiates those patients with both diseases from those with persistent inflammatory bowel disease who do not develop IgAN?

In this context, questions remain regarding the factors involved in the production of IgA and the possibility that plasma cells producing pathogenic IgA1 may not always originate from the intestinal mucosa. For example, experimental and clinical data linking tonsillitis and IgAN indicate involvement of tonsillar IgA-producing cells (22). B cells induced by nasopharyngeal-associated lymphoid tissue (NALT) may not only be homing to NALT and bronchus-associated lymphoid tissue (BALT), but also to other mucosal and lymphoid tissues that are involved in immune responses (23, 24). The “Mucosa-Bone marrow axis” hypothesis was proposed in the 1980s (25), based on the presence of polymeric IgA1-containing complexes in the serum and glomerular immunodeposits of patients with IgAN. Polymeric IgA is thought to be produced mainly by plasma cells in the mucosal tissues near the mucosal surfaces. The “Mucosa-Bone marrow axis” hypothesis postulates that nephritis-inducing IgA1-producing cells are induced in the mucosa and then migrate into the bone marrow to produce pathogenic IgA1 that initiates and sustains the disease. This hypothesis is supported by the finding that J-chain-containing IgA1-producing plasma cells are elevated in the bone marrow of IgAN patients (26).

IgA1 accounts for 85%-90% of serum IgA and is also produced by plasma cells (PCs) in the different tissues, including intestine, lungs, tonsils and nasal mucosa, whereas IgA2 is predominant in the colonic mucosa (27). Mucosa-associated bacteria often produce IgA-specific proteases that cleave amino acids in the hinge region of IgA1. In contrast, IgA2 is resistant to such cleavage by most of these proteases due to the differences in the hinge-region amino-acid sequence (28). Compared to IgA1, IgA2 activates neutrophils and macrophages more potently, which is thought to aid in efficient defense against infection (5). Conversely, it is IgA1 subclass that is involved in the formation of pathogenic immune complexes (29), and it is speculated that B cells induced in the NALT, including the tonsils, are involved.

It should be noted that clinical surveys of clinical presentation of IgAN in Europe and Japan indicated differences in the association of gastrointestinal complications with IgAN: for 17% of IgAN patients in Europe compared to 1% in Japan. Conversely, no significant difference in the incidence of macroscopic hematuria associated with upper-respiratory-tract infections was observed for IgAN patients in Japan (29.8%) *vs*. Europe (22.7%) (30).

## 2 IgAN, Gd-IgA1, and tonsils

IgAN is the most common primary glomerulonephritis in the world, with kidney failure occurring in most patients (31, 32). Considering that polymeric IgA1 is predominantly produced in mucosal tissues and a common clinical feature of IgAN is macroscopic hematuria associated with upper-respiratory tract infection, a possible link between the mucosal immune system and IgAN has been proposed (33, 34). Moreover, it is also not uncommon for urinary-tract abnormalities to occur after tonsillar irritation (35).

Subsequently, a search for IgAN-linked bacterial, viral, and/or food antigens has been undertaken, only to find no such generalized IgAN-specific agents (1, 36). Based on the accumulated data about the IgA1-containing immune complexes in the circulation of IgAN patients, biosynthesis of galactose-deficient IgA1 (Gd-IgA1), and IgG autoantibodies specific for Gd-IgA1 (37–44), the “multi-hit hypothesis” was proposed in 2011. This hypothesis on the pathobiology of IgAN has been widely accepted; it postulates that Gd-IgA1 glycoforms are bound in an immune complex with Gd-IgA1-specific IgG autoantibodies (45). Additional proteins are added and some of these complexes deposit in the glomeruli, causing kidney damage; serum levels of Gd-IgA1 and IgG autoantibodies are associated with disease progression (44, 46–48). Studies with cultured primary human mesangial cells showed that immune complexes consisting of IgA1 immune complexes (IgA1-IC) of molecular mass greater than 700 kDa induce cellular proliferation. Furthermore, the IgA1-IC isolated from serum of patients with active disease exhibiting macroscopic hematuria induced cellular proliferation of cultured mesangial cells to a higher degree than did the IgA1-IC isolated from sera of the same patients during a period of quiescent disease (40). Similarly, cultured mesangial cells incubated with serum IgA1 from IgAN patients activate extracellular signal-regulated kinase (ERK) of the mitogen-activated protein (MAP) kinase family, non-receptor type tyrosine kinase spleen tyrosine kinase (Syk) activation, and, subsequently, elevate production of proinflammatory cytokines and components of extracellular matrix (49–51).

**Figure 2:**
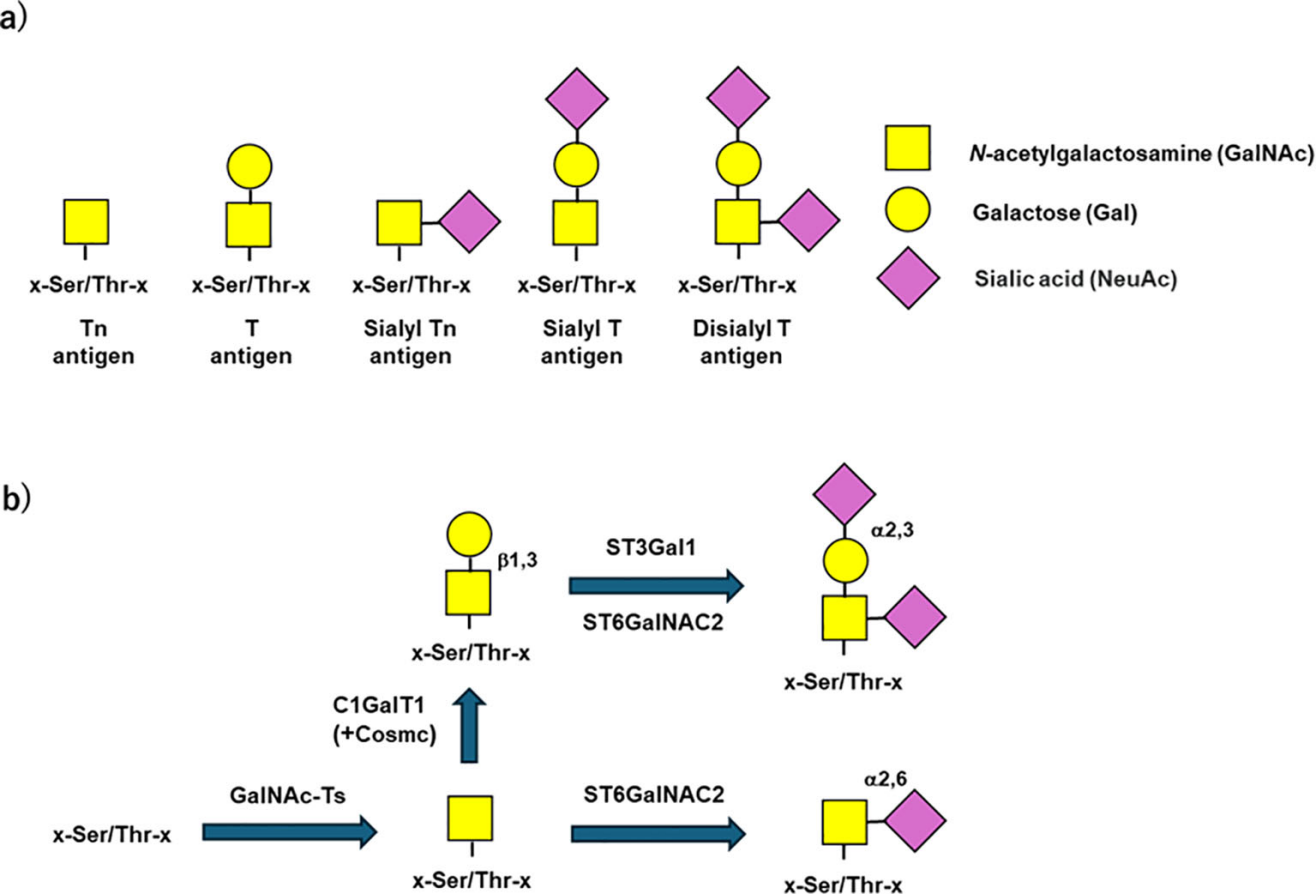
*O*-glycosylation of human IgA1. **(a)***O*-linked glycans of circulatory IgA1 are diverse in terms of their number, attachment sites, and composition. The Tn antigen (*N*-acetylgalactosamine; GalNAc) is usually modified by a specific galactosyltransferase (T-synthase, C1GalT1) in the Golgi apparatus. **(b)** IgA1 *O*-glycosylation pathways. The stepwise process begins with the attachment of GalNAc to some of the Ser/Thr residues in the hinge region catalyzed by a GalNAc-transferase(s) (GalNAc-T). GalNAc residues can be then modified by addition of Gal, mediated by core 1 β1,3- galactosyltransferase (C1GalT1). Production of the active C1GalT1 enzyme depends on its chaperone (C1GalT1C1, Cosmc). The core 1 structures (GalNAc-Gal) of IgA1 can be further modified by sialyltransferases that attach sialic acid to Gal (mediated by an ST3Gal enzyme, e.g., ST3Gal1) and/or GalNAc residues (mediated by an ST6GalNAc2 enzyme). Sialylation of GalNAc is mediated by ST6GalNAc2, as ST6GalNAc1 is not expressed in IgA1-producing cells (5). Conversely, if terminal GalNAc is sialylated by ST6GalNAc2, this structure cannot be further modified. Abnormal glycosylation of IgA1 is associated with dysregulated expression/activities of specific glycosyltransferases in IgA1-producing cells in patients with IgAN (42).

Biochemical studies using IgA1-secreting cell lines derived from peripheral blood of IgAN patients and healthy controls revealed that Gd-IgA1 production is driven by altered biosynthetic pathways of IgA1 *O*-glycans (52). The clustered *O*-glycans of circulatory IgA1 are diverse in terms of their numbers per hinge region, attachments sites, and composition (**Figure 2a**). Biosynthesis of IgA1 *O*-glycans occurs in the Golgi apparatus of IgA1-producing cells (**Figure 2b**). IgA1-secreting cell lines from IgAN patients showed reduced expression of *C1GALT1*, its chaperone *C1GALT1C1*, and increased expression of *ST6GALNAC2* (42). These gene-expression changes are associated with reduced Gal content in IgA1 secreted by IgA1-producing cells in IgAN patients (42). Notably, Gd-IgA1 in the circulation and glomerular immune-complex deposits of IgAN patients appears to be mainly in the polymeric form. Genetic studies showed that serum levels of Gd-IgA1 are genetically co-determined and GWAS identified genetic variations that impact the expression of *C1GALT1* and *C1GALT1C1* genes (53, 54). Moreover, expression of the glycosyltransferase C1GalT1 and the chaperon C1GalT1C1 can be altered by some pro-inflammatory cytokines (55–57).

Although some of the biochemical pathways involved in the production of Gd-IgA1 have been determined, the origin and locations of the IgA1-producing cells secreting polymeric Gd-IgA1 remain to be clarified. Although the gut mucosa has abundance of IgA1-producing cells, tonsillar B cells have also been proposed as a possible source of Gd-IgA1 (58). When considering the role of palatine tonsils in the pathogenesis of IgAN in general and Gd-IgA1 production in particular, we have to consider several concepts and ask various questions. For example, what factors affect the supply of circulatory IgA1 and Gd-IgA1 by the cells residing in tonsils? Are those Gd-IgA1-producing cells generated in the tonsils and then migrate to different locations? How are these cells maintained and renewed? In the sections below, we are discussing some of these points and evaluate the current knowledge and hypotheses within the framework of the genetic and geographic factors impacting disease heterogeneity.

## 3 Palatine tonsils as secondary lymph nodes in upper-respiratory tract mucosal immunity

The palatine tonsils, together with the pharyngeal tonsils, Eustachian tube tonsils, lingual tonsils, and pharyngeal lateral wall lymph follicles, are lymphoid tissues that with an annular arrangement in the pharynx are collectively known as the Waldeyer’s tonsil ring. The palatine tonsils belong to the mucosa-associated lymphoid tissue (MALT) and are involved in the primary immune response to airborne and gastrointestinal pathogens introduced via the oral or nasal cavity, resulting in antibody production (59–61). The palatine tonsils, the major organ of the NALT, have four specialized tissue compartments—namely, reticulo-epithelium, extrafollicular region, mantle zone of lymph follicles, and follicular germinal center (GC)—that take part in the immune functions (59). The palatine tonsils have B-lymphocyte-dominant lymphocytes and a small number of myelomonocytic cells. Palatine tonsils do not have an afferent lymphatic network as other lymphoid organs (e.g., lymph nodes). Human palatine tonsils have approximately 15 crypts that increase the surface area to enable access of antigens to lymphoid tissue after passage through a specialized epithelium. Recent RNA-seq analyses have revealed that the palatine tonsils represent a highly specialized form of mucosa-associated lymphoid tissue that is distinct from other secondary lymphoid organs. In particular, reticular cells within the tonsils exhibit unique transcriptional profiles and spatial organization adapted to the environment of persistent antigen exposure at the oropharyngeal mucosa, and have been shown to play a critical role in the local regulation of T-cell and B-cell immune responses (62, 63). Dendritic cells take up exogenous antigens and transport them to extrafollicular T-cell areas and B-cell follicles (64). Antigen-presenting naive B cells are activated in the extrafollicular region. Some of these B cells undergo clonal proliferation, somatic hypermutation, affinity maturation, immunoglobulin class switching, and finally differentiate into plasma cells or memory B cells (59). Palatine tonsils undergo morphological, histopathological, and immunological changes with age: the size of palatine tonsils is prominent in childhood but decreases with age (65, 66). Histopathologically, parenchymal and lymph follicular areas decrease with age, while fibrous connective tissue, collagen fibers and elastic-fiber areas increase with age (67). Immunologically, the proportion of germinal center B cells decreases with age and the proportion of memory B cells increases with age. Ig isoforms preferentially switch from IgM to IgA with age (68).

Tonsillar lymphocytes proliferate *ex vivo* and synthesize DNA even when cultured in the absence of mitogens; B cells exhibit a high-production capacity for IgG and IgA (69, 70). These findings indicate that, unlike the peripheral-blood and peripheral-lymph-node lymphocytes, the tonsillar lymphocytes are enriched for activated B cells.

Moreover, the immune responses of the tonsils are aimed against pathogens and do not respond to tonsil-resident bacteria, such as alpha streptococci *Streptococcus sanguinis*, *S. salivarius*, and *S. mitis*, due to the immune-tolerance mechanisms (71). However, a DNA sequence common to all bacteria, unmethylated CpG-oligodeoxynucleotide (CpG-ODN) is a ligand for TLR9 that induces an innate immune response. TLRs mediate signaling to lymphocytes through inflammatory cytokines and maturation of dendritic cells, thus playing important roles in mucosal immunity (72, 73). Activation of the innate immune system may increase the production of nephritogenic IgA, independent of the specific antigen. Nasal stimulation in a mouse model of spontaneous IgAN with the TLR9 ligand CpG-ODN leads to exacerbated renal damage and increase in serum IgA levels and IgA mesangial deposits. The severity of glomerular damage in this model is related to the degree of TLR9 expression in splenocytes (74). In the tonsils of IgAN patients, immune tolerance is disrupted and there may be an excessive immune response to indigenous bacteria and their antigens, including bacterial DNA. These findings in a mouse model raise the question whether TLR9 may be involved in the pathogenesis of human IgAN, as discussed below.

## 4 Contribution of tonsillar B cells to circulatory IgA in IgAN

As noted before, polymeric serum IgA1 with aberrant *O*-glycosylation is implicated in the pathogenesis of IgAN as an autoantigen (45), and serum levels of Gd-IgA1 predict disease progression in IgAN (46, 48). In connection with the role of tonsillar cells in IgA production, it was reported that serum IgA levels decreased after tonsillectomy in patients with IgAN (75, 76). Furthermore polymeric-IgA-producing cells are elevated in the tonsils of IgAN patients (77), and cultured mitogen-stimulated tonsillar lymphocytes exhibit enhanced production of polymeric IgA (78). B-1 cells (CD19+,CD5+) may be the main type of IgA-producing cells in mucosal tissues (79), and these cells are increased in the germinal centers of tonsils of most IgAN patients (80). Notably, several studies reported that some but not all patients improved clinically in terms of reduced serum levels of IgA after tonsillectomy (81, 82); the patients who did not improve clinically after tonsillectomy did not have increased B-1 cells in the tonsillar GCs. IgA antibodies derived from B-1 cells against commensal bacteria are not natural antibodies but are specifically induced by antigenic stimulation. This also suggests that this pathway is independent of T cells and the organization of follicular lymphoid tissues (12, 83). It thus will be important to determine the role of B-1 cells in IgAN and their contribution to serum IgA in patients with IgAN.

Intra-tonsillar vaccination with tetanus vaccine induced IgG- and IgA-secreting cells; a fraction of B cells activated in tonsils entered the circulation and disseminated to distant organs and vaccine-specific antibodies were subsequently detected in serum and pharyngeal secretions (84). This and other studies thus demonstrated that tonsils could serve as inductive sites for immune responses in the upper-respiratory tract with physiological as well as pathophysiological implications (85–89).

Furthermore, elevated carriage of *Neisseria* species in the tonsils and increased presence of serum anti-Neisseria IgA was found in IgAN patients (90). Furthermore, transgenic mice with human B-cell activating factor (BAFF-Tg mice) after nasal infection with *Neisseria* developed elevated serum levels of anti-Neisseria IgA, and anti-Neisseria IgA-secreting cells were found in the kidneys. This finding indicates that IgA-producing cells induced by exogenous antigen exposure in the airways may migrate to other sites, including the kidneys (90). Although the functional capacities of these B cells are not fully understood, it is likely that they cells undergo a maturation process during the GC response.

The IgA1 circulating in the blood and deposited in the glomeruli of patients with IgAN is enriched for aberrantly glycosylated IgA1, Gd-IgA1. Gd-IgA1 is also produced by tonsillar mononuclear cells, and the tonsils may be an important site of Gd-IgA1 production in some patients (91, 92). In addition, IL-6 or leukemia inhibitory factor (LIF) stimulate the tonsil-derived IgA1-producing cell lines from IgAN patients, as well as their peripheral-blood-derived IgA1 cells, to overproduce Gd-IgA1 due to abnormal signal transducer and activator of transcription 3 (STAT3) or signal transducer and activator of transcription 1 (STAT1) signaling (93–95). Elevated activation of STAT1 in the peripheral-blood mononuclear cells and kidney tissues of IgAN patients is associated with proteinuria but not with disease progression (96).

Activation of TLRs can induce a class switch of B cells to IgA in the absence of T cells via cytokines, such as BAFF and a proliferation-inducing ligand (APRIL) (97). In IgAN patients, increased APRIL expression in tonsillar GCs is associated with increased TLR9 expression and with worse urinary findings. TLR9 involvement in the pathogenesis of IgAN may be mediated via the APRIL pathway, leading to plasma-cell maturation (22). Stimulation of tonsillar mononuclear cells from patients with IgAN with CpG-ODN induces T cell-independent production of BAFF, IFN-γ, and IgA (98). BAFF increases Bcl-2 expression in B cells, that inhibits B cell apoptosis (99). Interestingly, Bcl-2 can increase the production of abnormally glycosylated IgA1 and promote renal glomerular IgA deposition (100). BAFF overproduction mediated by CpG-ODN in tonsils of IgAN patients may be involved in qualitative as well as quantitative abnormalities of IgA1.

In clinical studies, TLR9 hyper-expression in tonsils is associated with higher efficacy of tonsillectomy combined with steroid-pulse therapy (101); reports of TLR9 gene polymorphism being associated with the pathogenesis of IgAN is in line with the proposed CpG-ODN-TLR9 pathway (74). Moreover, expression of TLR7 in tonsils is upregulated in IgAN patients and correlates with the expression of APRIL, adding another TLR-mediated pathway to the list (102). In addition, increased expression of various TLRs in peripheral-blood monocytes has been observed in patients with IgAN, indicating a chronic activation of innate immunity in IgAN patients (103, 104).

## 5 Tonsillectomy as a treatment for IgAN

In Japan, the combination of tonsillectomy and steroid-pulse therapy has frequently resulted in clinical remission (defined by negative results for urinary occult blood and protein), and this therapy is commonly used for Japanese IgAN patients (105–107). To investigate the possible association between tonsillectomy and outcomes in patients with IgAN, a multicenter analysis of 1,065 patients (with a mean follow-up time of 5.8 years) was performed with a primary endpoint of 1.5-fold increase in serum creatinine and initiation of dialysis; propensity-score matching showed a 57-66% risk reduction in the tonsillectomy group compared to the no-tonsillectomy group (108). With these results, The Kidney Disease: Improving Global Outcomes (KDIGO) 2025 guidelines (https://www.sciencedirect.com/journal/kidney-international/vol/108/issue/4/suppl/S) have been revised and indicate that tonsillectomy should be considered in Japan on an individual-case basis. In examining predictors, tonsillectomy and/or corticosteroids therapy in patients with eGFR <60 mL/min/1.73 m^2^ and urinary protein <0.5 g/day tended to reduce cumulative outcome incidence compared to other types of treatment, i.e., not including corticosteroids and tonsillectomy (109). In addition, a report from a randomized prospective study (J-IGACS) showed that adding tonsillectomy to systemic corticosteroid therapy was associated with a 60% risk reduction of renal events in IgAN patients (110). Notably, no long-term negative effects of tonsillectomy on immune function have been observed (111, 112).

In the clinical context, it is well known that in patients with IgAN, tonsillectomy decreases serum IgA levels, with an average reduction of about 10% in one study (101). Comparison of groups with greater *vs*. smaller reduction in serum IgA levels after tonsillectomy revealed a possible association with TLR9 – patients with high TLR9 expression in the tonsils exhibited greater reduction of serum IgA levels after tonsillectomy (101). This observation is in line with the hypothesis that some of the nephritis-inducing IgA1 (i.e., Gd-IgA1) may be related to tonsillar B cells and involve TLR pathways. In another study, IgAN patients who had Gd-IgA1 serum levels reduced after tonsillectomy exhibited a greater reduction of hematuria compared to the patients whose serum Gd-IgA1 were not reduced after tonsillectomy. Moreover, tonsillar TLR9 expression levels were high in IgAN patients who exhibited reduction in serum Gd-IgA1 levels after tonsillectomy (58). Furthermore, tonsillectomy performed in the first year after renal transplantation reduced the level of serum Gd-IgA1 and the recurrence rate of histological IgAN (113). These findings suggest that serum Gd-IgA1 from tonsillar B cells may play an important role in the development of IgAN.

With respect to the multi-hit hypothesis for IgAN, are any of these observations relevant to the production of IgG autoantibodies specific for Gd-IgA1? Given the variation in the glycosylation of the hinge region of IgA1 and of the cloned IgG antibodies, it is likely that immune complexes are formed by polyclonal IgG autoantibodies rather than by an autoantibody produced by a single clone of B cells (27). What is the localization of the cells producing these autoantibodies that drive the formation of pathogenic immune complexes? Could tonsils be among the sites that harbor the cells producing these IgG autoantibodies? Are these autoantibodies formed in a T cell-dependent fashion? Interestingly, an increase in the number of B-1 cells in the tonsil GC was observed in IgAN patients who showed clinical improvement after tonsillectomy. In contrast, no increase in B-1 cells in tonsil GCs was observed in patients who did not improve after tonsillectomy (80). Furthermore, as B-1 cells are increased in the peripheral blood of patients with IgAN (114), it is possible that B cells involved in production of Gd-IgA1 and/or IgG autoantibodies may also migrate to or from the tonsils.

## 6 IgA, tonsillectomy, and IgAN: findings from GWAS and signaling studies

**Figure 3:**
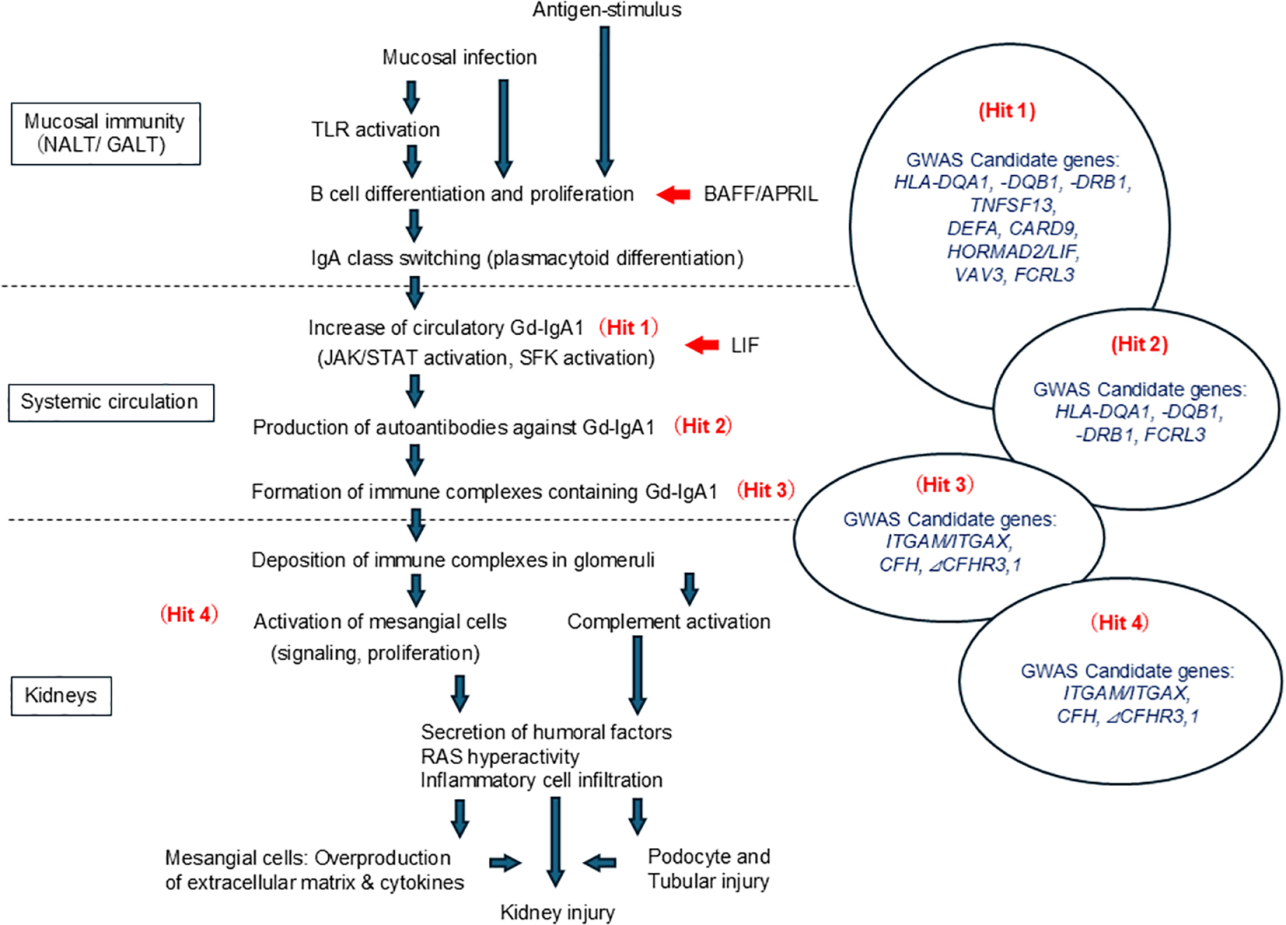
Pathogenesis of IgA nephropathy (IgAN) and the associated IgAN-susceptibility genetic loci. IgAN is thought to involve immune-system stimulation by foreign antigens related to the upper-respiratory tract and gastrointestinal tract infections. These processes involve T cell-dependent and independent pathways enhancing the production of Gd-IgA1, with baseline-Gd-IgA1 production being genetically co-determined. APRIL and BAFF enable isotype switch in B cells, leading to plasmacytoid differentiation and elevated Gd-IgA1 production that can be also enhanced by IL-6 and LIF due to abnormal JAK/STAT signaling (Hit 1). In genetically susceptible individuals, autoantibodies specific for Gd-IgA1 are produced (Hit 2), driving production of immune complexes that have additional proteins added (Hit 3). Some of these complexes deposit in the glomeruli, activate mesangial cells, leading to kidney injury (Hit 4). Various humoral factors produced by the activated mesangial cells also induce podocyte injury and tubulointerstitial damage, and contribute to the development of nephritis. NALT, Nasal-associated lymphoid tissue; GALT, Gut-associated lymphoid tissue; TLR, Toll-like receptor; BAFF, B-cell activating factor belonging to the Tumor-Necrosis Family; APRIL, A proliferation-inducing ligand; Gd-IgA1, Galactose-deficient IgA1; JAK/STAT, Janus kinase/signal transducer and activator of transcription; SFK, Src-family kinase; RAS, Renin-Angiotensin-Aldosterone System; GWAS, genome-wide association studies; *HLA-DQA1*, major histocompatibility complex, class II, DQ α 1; *HLA-DQB1*, major histocompatibility complex, class II, DQ β 1; *HLA-DRB1*, major histocompatibility complex, class II, DR β 1; *FCRC3*, Fc receptor like 3; *TNFSF13*, Tumor Necrosis Factor Superfamily, Member 13; *DEFA*, Defensin α; *CARD9*, Caspase recruitment domain-containing protein 9; *HORMAD2*, HORMA Domain Containing 2; *LIF*, Leukemia inhibitory factor; *VAV3*, Vav Guanine Nucleotide Exchange Factor 3; *ITGAM/ITGAX*, Integrin α M/Integrin α X; *CFH*, Complement factors H; *CFHR3,1*, Complement Factor H Related 3,1.

GWAS of IgAN in patients of Asian and European ancestry revealed multiple candidate genes with many of those known to participate in regulation of mucosal immune responses (15, 115–124). The latest GWAS revealed 30 loci associated with the risk of IgAN (125). Among the candidate genes were those that regulate mucosal-associated lymphoid tissues involved in IgA production, such as *ITGAM* and *TNFSF13* (**Figure 3**). From the top 26 high-priority ‘biological candidates’, 11 can be targeted directly or indirectly by existing drugs. Some candidate drugs are in various clinical trials. These drugs include: (a) inhibitors of the alternative or lectin complement pathway, currently in clinical trials for different types of glomerulopathies (126, 127). The complement targets applicable to IgAN include MASP-2, complement factors B and D, C3, C5, and C5a receptors 1 (C5aR1) (128). (b) Drugs targeting B cells by inhibiting APRIL or its receptors, e.g., transmembrane activator and calcium modulator and cyclophilin ligand interactor (TACI), are already in clinical trials for IgAN. A recent clinical trial reported that administration of TACI-IgG Fc fusion protein (Atacicept), an inhibitor of APRIL, to IgAN patients reduced serum levels of Gd-IgA1 and improved proteinuria (129). Similarly, a humanized IgG2 monoclonal antibody (Sibeprenlimab), a neutralizing antibody for APRIL, has also been reported to reduce serum levels of Gd-IgA1 and improve proteinuria (130). Unfortunately, most of the IgAN-associated causal genes with the risk alleles increasing the gene expression, such as *CARD9*, *ITGAX*, *PF4V1*, *CFHR1* or *FCAR*, do not yet have effective drug inhibitors (125).

*HORMAD2* IgAN-associated locus contains multiple genes, including *LIF* and oncostatin M (*OSM*). This locus is also associated with tonsillectomy and IgA serum levels (124, 131) and its biological effects may include TLR9 pathways (132). Notably, *LIF* is a high-scoring drug-target gene (125). The cytokines LIF and IL-6, when bound to receptor complexes comprising gp130/LIF receptor and gp130/IL-6 receptor, respectively, activate JAK/STAT pathways and alter the expression of several genes (57).

In the IgA1-producing cell lines derived from peripheral blood of patients with IgAN, aberrant STAT1 signaling mediated by LIF was associated with Gd-IgA1 overproduction. Moreover, kinomic studies revealed that baseline activities of several protein tyrosine kinases (PTKs) in IgA1-producing cells from IgAN patients were higher than those in cells from healthy controls (HC). Most of these PTKs were associated with growth factors Eph and Src, signaling pathways related to cell growth, transcription factors, and cross-activation of PTK receptors. Furthermore, LIF activation further increased activities of PTKs in the Src family in IgA1-producing cells from IgAN patients but not in the cells from HC. Conversely, in the cells from HC, LIF activated fibroblast growth factor receptors (FGFR), platelet-derived growth factor receptor (PDGFR) and epidermal growth factor receptor (EGFR) systems (94).

Whereas LIF signaling in IgA1-secreting cells derived from peripheral blood utilizes STAT1, IL-6 signaling exclusively uses STAT3. Furthermore, IgA1-secreting cells from peripheral blood from patients with IgAN are abnormally activated by IL-6 compared to those from HC. Specifically, IL-6 induced enhanced and prolonged phosphorylation of STAT3 in the cells from patients with IgAN, a process that results in overproduction of Gd-IgA1. Analysis of nine independent STAT3 ChIP-seq datasets derived from B cells identified multiple STAT3-binding sites located both upstream and downstream of the *C1GALT1* locus, a key glycosyltransferase gene encoding enzyme involved in galactosylation of IgA1 *O*-glycans. These findings provide further insight into a potential regulatory mechanism of *C1GALT1* mediated by IL-6/STAT3 signaling. Notably, transcription of *C1GALT1* is known to be critically dependent on SP1/3 binding to its promoter region (133). In this context, activated STAT3 may exert a negative regulatory effect by interfering with SP1/3-mediated transcriptional activity. Such a mechanism is consistent with previous observations demonstrating downregulation of *C1GALT1* transcription following IL-6 stimulation (56). This IL-6-mediated overproduction of Gd-IgA1 is inhibited by Stattic, a specific STAT3 inhibitor, and AZD1480, a JAK2 small molecule inhibitor, in a dose-dependent manner (93).

As B cells from the peripheral blood may differ in many characteristics from those in lymphatic tissues, we expanded our studies to include IgA1-producing cells from the palatine tonsils. Using IgA1-producing cell lines derived from the tonsils of IgAN patients and disease controls (obstructive sleep apnea), we determined that LIF stimulation leads to Gd-IgA1 overproduction, but only in the tonsillar cell lines of IgAN patients (95). This LIF-induced Gd-IgA1 overproduction in IgAN-derived tonsillar cells was mediated by STAT1, as confirmed by STAT1 siRNA knock-down. Moreover, a JAK2 inhibitor, AZD1480 exhibited a dose-dependent inhibition of the LIF-induced Gd-IgA1 overproduction, further confirming that LIF utilizes JAK2/STAT1 signaling pathway. Unexpectedly, high concentrations of AZD1480, but only in the presence of LIF, reduced Gd-IgA1 production in the cells derived from patients with IgAN to that of the control cells from patients with obstructive sleep apnea. Although IL-6 also induced overproduction of Gd-IgA1 in the IgA1-producing cell lines derived from the tonsils of IgAN patients, JAK2 inhibitors did not reduce overproduction of Gd-IgA1 to that of controls.

In summary, IgA1-producing cells from patients with IgAN exhibit abnormal responses to at least two cytokines of the IL-6 family: IL-6 and LIF. Each of these two cytokines uses a different STAT protein for signal transduction: IL-6 uses STAT3 whereas LIF signals are mediated by STAT1. Moreover, IgA1-producing cells lines derived from tonsils *vs*. the circulation exhibit differences in LIF/LIFR/JAK2/STAT1 signaling pathways, underscoring differential characteristics of IgA1-secreting cells from peripheral blood *vs*. lymphatic tissues. Moreover, identifying the cell populations upstream of the IL-6/LIF signaling pathway as well as the microenvironment that drives Gd-IgA1 production—particularly the interactions between stromal cells and immune cells within mucosa-associated lymphoid tissue—represent critical challenges for follow-up studies.

Further studies will need to elucidate how these functional differences may impact IgAN and what implications there may be for the use of tonsillectomy in Japan with respect to the time interval from disease onset/diagnosis.

## 7 Conclusion

**Figure 4:**
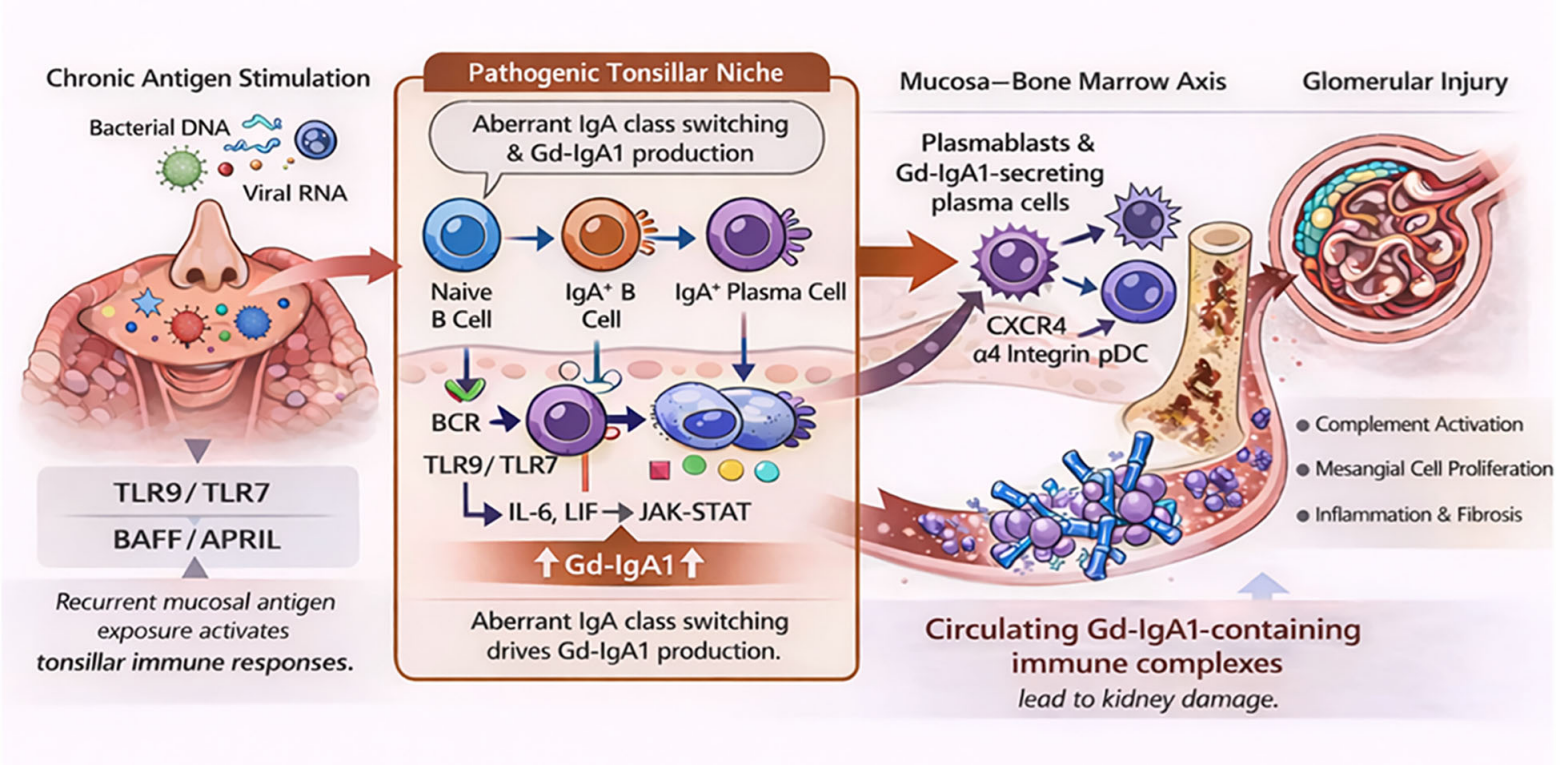
Cellular and molecular mechanisms of tonsillar mucosal immunity contributing to the pathogenesis of IgA nephropathy (IgAN). This schematic illustrates how chronic mucosal antigen stimulation in the tonsils leads to the generation of pathogenic IgA responses and subsequent glomerular injury in IgAN in susceptible individuals. In the left panel, recurrent exposure to mucosal antigens, such as bacterial DNA (CpG) and viral RNA, activates TLR9/TLR7 signaling and BAFF/APRIL pathways in the tonsils, initiating local immune responses. The central panel depicts a pathogenic tonsillar niche, in which B-cell receptor (BCR) signaling cooperates with TLR9/TLR7 activation, leading to the induction of IL-6– and LIF-mediated JAK–STAT signaling. These pathways drive aberrant IgA class switching and increased production of galactose-deficient IgA1 (Gd-IgA1). Naive B cells differentiate into IgA^+^ B cells and IgA^+^ plasma cells, resulting in amplification of pathogenic IgA responses. The second panel from the right illustrates the hypothesis on mucosa–bone marrow axis, whereby Gd-IgA1–producing plasmablasts migrate to the bone marrow through CXCR4 and α4β1 integrin–dependent homing mechanisms, establishing a sustained source of circulating Gd-IgA1. However, Gd-IgA1-producing cells may also migrate to other tissues, including the lungs and intestine. In the far-right panel, circulating Gd-IgA1-containing immune complexes deposit in the glomerular mesangium, inducing mesangial cell proliferation, matrix expansion, and production of pro-inflammatory cytokines, ultimately resulting in glomerular injury. This schematic figure was created using an AI-assisted image generation tool (ChatGPT, OpenAI) and were subsequently edited by the authors.

In this review, we have described mucosal immune abnormalities in IgAN with an emphasis on the upper-airway mucosa and the origins and pathways leading to the production of aberrantly glycosylated IgA1, Gd-IgA1 (**Figure 4**). Multiple findings collectively suggest that these pathogenic glycoforms of IgA1 are produced by IgA1-secreting cells originating from or residing in mucosal tissues, e.g., gut and tonsils, and that some growth factors and cytokines activate these cells and induce overproduction of Gd-IgA1. NALT and gut-associated lymphoid tissue (GALT) are the main sites with IgA-producing cells and both may exhibit abnormal immune responses relevant to IgAN (134). For example, in experimental model systems of intestinal mucosa, loss of STAT3 signaling disrupted the mucosal barrier, causing differentiation of cells forming the progenitor cell niche and abnormal proliferation of progenitor cells (135). Furthermore, aberrant LIF-mediated STAT1 signaling in tonsillar cells enhances Gd-IgA1 production. These phenomena, together with GWAS data, provide clues that will lead to elucidating the origin of IgA1-producing cells and pathological mechanisms of IgAN.
